# Surgical Occlusion Setup in Correction of Skeletal Class III Deformity Using Surgery-First Approach: Guidelines, Characteristics and Accuracy

**DOI:** 10.1038/s41598-018-30124-2

**Published:** 2018-08-03

**Authors:** Yu-Fang Liao, Shu Hsien Lo

**Affiliations:** 1 Department of Craniofacial Orthodontics, Chang Gung Memorial Hospital, Taoyuan, 333 Taiwan; 2 Graduate Institute of Dental and Craniofacial Science, College of Medicine, Chang Gung University, Taoyuan, 333 Taiwan; 3 Craniofacial Research Center, Chang Gung Memorial Hospital, Linkou, 333 Taiwan; 4 Department of Craniofacial Orthodontics, Chang Gung Memorial Hospital, Linkou, 333 Taiwan

## Abstract

The aims of this study were to establish guidelines for the surgical occlusion setup of surgery-first orthognathic surgery, and evaluate the resulting characteristics and accuracy. Skeletal Class III patients (N = 53) underwent Le Fort I osteotomy and bilateral sagittal split osteotomy. Study models before orthognathic surgery were set according to the guidelines. Occlusion was measured and computer-aided surgical simulation was used to evaluate the characteristics and accuracy of the surgical occlusion. The mean age of participants was 25 ± 6 years with 24 males and 29 females. The occlusion was set as positive overjet (4.4 ± 2.0 mm) and overbite (1.4 ± 1.8 mm), Class II or I molar relation, and posterior cross bite (overjet: 4.9 ± 2.0 mm and 4.4 ±1.9 mm, respectively for the right and left second molars) and open bite (overbite: −2.0 ± 1.6 mm and −1.9 ± 1.3 mm, respectively for the right and left second molars). Normal jaw relationship and symmetry were noted after virtual surgery. None of the patients required new occlusal setup. Our data contribute the use of the surgery-first approach for skeletal Class III patients by establishing guidelines for a surgical occlusion setup in three dimensions.

## Introduction

Before the 1960s, most orthognathic surgeries were performed either without orthodontic treatment after removing the orthodontic appliances, or before any orthodontic treatment. Later, the three stages of classic surgical orthodontic treatment became popular because of the stability of the results and satisfaction with the post-treatment outcomes^1^. The 3-stage approach requires a variable length of presurgical orthodontic preparation to decompensate the malocclusion, which is followed by surgical correction of the skeletal discrepancy and a relatively short period of postsurgical orthodontics for detailing and finishing of the occlusion. Presurgical orthodontics typically includes dental alignment, incisor decompensation, arch leveling and coordination, and usually requires 15 to 24 months^2,3^. However, this exacerbates facial esthetics and dental function, and causes significant patient discomfort before surgery^2^. One study found that one third of patients rated the orthodontics as the worst part of their orthognathic treatment owing to the appliances’ visibility and discomfort, and the length of treatment^4^.

The longer treatment time and transitional detriment to the facial esthetics and dental function associated with presurgical orthodontics have led to a new approach called “surgery-first”, which eliminates the presurgical orthodontic phase^5–9^. The orthognathic surgery-first approach is becoming popular because of several advantages such as reduced treatment time, efficient tooth decompensation, and early improvement in facial esthetics, especially in Class III malocclusion^5,6,8–10^. These advantages have a very positive influence on patients’ global satisfaction with treatment.

The most difficult step for the surgery-first approach is the setup of the transitional occlusion at the time of surgery (i.e., surgical occlusion). There are some reports showing guidelines for surgical occlusion setup for surgery-first approach in Class III malocclusion^7,8,11–13^. However, the guidelines are rather crude; that is, dental occlusion is proposed in the antero-posterior dimension only^8,11,13,14^ and, in addition, there are no data available on the occlusal characteristics or accuracy. Accurate surgical occlusion setup is important to avoid severe postoperative occlusal instability, incomplete or excessive skeletal correction, or skeletal asymmetry (i.e., skeletal deformity). Currently, 3D virtual simulation process allows us to assess the accuracy of occlusion setup in terms of skeletal deformity. Thus, we conducted this study to establish guidelines for surgical occlusion setup, and to investigate the characteristics and accuracy of the surgical occlusion.

## Results

The 53 patients enrolled in the study were comprised of 24 males and 29 females; mean age was 25 ± 6 years. Genioplasty was performed on 39 patients (Table 1). Thirty-nine patients (74%) had occlusal contact on three segments. The average number of tooth contact was 5.2 ± 2.3 (Table 2). Positive overjet (mean 4.4 ± 2.0 mm) and overbite (mean 1.4 ± 1.8 mm), Class II or I molar relation, and posterior cross bite (buccal overjet: mean 4.9 ± 2.0 mm and 4.4 ±1.9 mm, respectively for the right and left molars) and open bite (buccal overbite: mean −2.0 ± 1.6 mm and −1.9 ± 1.3 mm, respectively for the right and left molars) on second molars were determined at setup (Table 3). Normal jaw relationship (ANB: mean 2.1 ± 1.8 degrees) and symmetry was noted after virtual surgery (Table 4). None of the patients required new occlusal setup due to significant skeletal deformity. There were no differences in characteristics and accuracy of surgical occlusion as well as jaw relation and symmetry after virtual surgery between patients who received genioplasty and those who did not receive genioplasty (Tables 1 through 4).

**Table 1:** Patients demographics and characteristics.

	Total Patients (N = 53)	With Genioplasty (N = 39)	Without Genioplasty (N = 14)	P
Female, n (%)	29 (55)	22 (76)	7 (50)	0.68^†^
Age at surgery, years (Mean ± SD)	25 ± 6	24 ± 5	27 ± 8	0.37^†^
ANB, degree (Mean ± SD)	−4.3 ± 3.0	−3.8 ± 2.7	−4.7 ± 2.7	0.30^†^
Overjet, mm (Mean ± SD)	−3.3 ± 3.7	−3.0 ± 3.4	−4.2 ± 4.4	0.31^†^
Overbite, mm (Mean ± SD)	−0.1 ± 3.1	0.1 ± 2.7	−0.6 ± 4.1	0.48^†^
**Right first molar relation**				
Angle Class I, n (%)	6 (11)	6 (15)	0 (0)	0.22^‡^
Angle Class II, n (%)	0 (0)	0 (0)	0 (0)	
Angle Class III, n (%)	41 (78)	28 (72)	13 (93)	
Not available, n (%)	6 (11)	5 (13)	1 (7)	
**Left first molar relation**				
Angle Class I, n (%)	3 (6)	3 (8)	0 (0)	0.63^‡^
Angle Class II, n (%)	1 (2)	1 (2)	0 (0)	
Angle Class III, n (%)	43 (81)	31 (80)	12 (86)	
Not available, n (%)	6 (11)	4 (10)	2 (14)	
Midline discrepancy, mm (Mean ± SD)	1.6 ± 1.2	1.6 ± 1.1	1.5 ± 1.3	0.70^†^

^†^Statistical analysis was carried out using the Mann Whitney U test (with versus without genioplasty).

^‡^Statistical analysis was carried out using the chi-square test (with versus without genioplasty).

**Table 2:** Occlusal contact of surgical occlusion.

Occlusal Contact	Total Patients (N = 53)	With Genioplasty (N = 39)	Without Genioplasty (N = 14)	P*
**Contact Distribution, n (%)**				
Three segments	39 (74)	31 (79)	8 (57)	0.17^‡^
Two segments	10^a^ (19)	5^c^ (13)	5^e^ (36)	
One segment	4^b^ (8)	3^d^ (8)	1^f^ (7)	
**Contact Amount, n**				
Anterior teeth (Mean ± SD)	1.7 ± 1.3	1.6 ± 1.1	1.9 ± 1.7	0.75^†^
Premolars (Mean ± SD)	1.8 ± 1.3	1.8 ± 1.3	1.7 ± 1.5	0.73^†^
Molars (Mean ± SD)	1.7 ± 1.2	1.7 ± 1.2	1.9 ± 1.1	0.34^†^
Total (Mean ± SD)	5.2 ± 2.3	5.1 ± 2.2	5.5 ± 2.5	0.57^†^

^a^7 posterior right and posterior left segments, 3 anterior and posterior right segments.

^b^3 anterior segment, 1 posterior left segment.

^c^3 posterior right and posterior left segments, 2 anterior and posterior right segments.

^d^2 anterior segment, 1 posterior left segment.

^e^4 posterior right and posterior left segments, 1 anterior and posterior right segments.

^e^1 anterior segment.

^†^Statistical analysis was carried out using the Mann Whitney U test (with vs. without genioplasty).

^‡^Statistical analysis was carried out using the chi-square test (with vs. without genioplasty).

**Table 3:** Occlusal characteristics of surgical occlusion.

Characteristics	Total Patients (N = 53)	With Genioplasty (N = 39)	Without Genioplasty (N = 14)	P
**Incisor Relation**				
Overjet, mm (Mean ± SD)	4.4 ± 2.0**^a^	4.1 ± 1.8**^a^	5.0 ± 2.5*^b^	0.25^†^
Overbite (Mean ± SD)	1.4 ± 1.8**^a^	1.2 ± 1.9**^a^	1.8 ± 1.6*^b^	0.43^†^
**Right second molar relation**				
Overjet, mm (Mean ± SD)	4.9 ± 2.1**^b^	4.9 ± 1.7**^b^	4.9 ± 2.9*^b^	0.68^†^
Overbite, mm (Mean ± SD)	−2.0 ± 1.6**^b^	−2.0 ± 1.4**^a^	−1.8 ± 2.0*^b^	0.97^†^
**Left second molar relation**				
Overjet, mm (Mean ± SD)	4.4 ± 1.9**^b^	4.6 ± 1.8**^b^	4.1 ± 2.1*^b^	0.62^†^
Overbite, mm (Mean ± SD)	−1.9 ± 1.3**^a^	−1.9 ± 1.4**^a^	−2.1 ± 1.2*^b^	0.44^†^
**Right first molar relation**				
Angle Class I, n (%)	18 (34)**^c^	12 (31)**^c^	7 (50)*^c^	0.50^‡^
Angle Class II, n (%)	25 (47)	19 (49)	5 (36)	
Angle Class III, n (%)	4 (8)	3 (7)	1 (7)	
Not available, n (%)	6 (11)	5 (13)	1 (7)	
**Left first molar relation**				
Angle Class I, n (%)	16 (30)**^c^	14 (36)**^c^	3 (22)*^c^	0.17^‡^
Angle Class II, n (%)	30 (57)	21 (54)	8 (57)	
Angle Class III, n (%)	1 (2)	0 (0)	1 (7)	
Not available, n (%)	6 (11)	4 (10)	2 (14)	
Midline discrepancy, mm (Mean ± SD)	0.8 ± 0.9**^b^	0.7 ± 0.7**^b^	1.1 ± 1.4*^b^	0.77^†^

^†^Statistical analysis was carried out using the Mann Whitney U test (with vs. without genioplasty).

^‡^Statistical analysis was carried out using the chi-square test (with vs. without genioplasty).

*p < 0.05 (before vs. after simulation).

**p < 0.001 (before vs. after simulation).

^a^Statistical analysis was carried out using the paired t-test (before vs. after simulation).

^b^Statistical analysis was carried out using the Wilcoxon signed ranks test (before vs. after simulation).

^c^Statistical analysis was carried out using the chi-square test (before vs. after simulation).

**Table 4:** Virtual outcome regarding jaw relation and symmetry.

Jaw Discrepancy	Total Patients (N = 53)	With Genioplasty (N = 39)	Without Genioplasty (N = 14)	P*
**Jaw relation**				
ANB, degree (Mean ± SD)	2.1 ± 1.8**^a^	2.4 ± 1.7**^a^	1.8 ± 1.5*^b^	0.34
**Jaw symmetry**				
ANS deviation, mm (Mean ± SD)	0.7 ± 0.8	0.7 ± 0.8	0.5 ± 0.9	0.11
Upper incisor deviation, mm (Mean ± SD)	0.5 ± 0.6	0.4 ± 0.6	0.5 ± 0.8	1.00
Lower incisor deviation, mm (Mean ± SD)	0.4 ± 0.7	0.3 ± 0.5	0.6 ± 1.2	0.64
Menton deviation, mm (Mean ± SD)	0.7 ± 0.8	0.7 ± 0.7	0.8 ± 1.1	0.72
Vertical body discrepancy, mm (Mean ± SD)	0.8 ± 0.5	0.8 ± 0.5	0.6 ± 0.5	0.13
Horizontal body discrepancy, mm (Mean ± SD)	1.9 ± 1.4	1.9 ± 1.4	1.7 ± 1.4	0.42
Volumetric body discrepancy, mm^3^ (Mean ± SD)	916.0 ± 562.7	945.1 ± 628.4	834.8 ± 320.9	0.78

ANS: anterior nasal spine.

*Statistical analysis was carried out using the Mann Whitney U test (with versus without genioplasty).

*p < 0.05 (before vs. after simulation).

**p < 0.001 (before vs. after simulation).

^a^Statistical analysis was carried out using the paired t-test (before vs. after simulation).

^b^Statistical analysis was carried out using the Wilcoxon signed ranks test (before vs. after simulation).

## Discussion

Despite the distinct advantages of the surgery-first approach, there has been no conclusive data regarding variables required for the surgical occlusion setup. To our knowledge, this is the first study to quantify the surgical occlusion setup for a surgery-first approach and subsequently evaluate the accuracy of the setup. We found that the majority of occlusion setups had contact on three segments, and the accuracy of the setup was excellent. Our data contribute the use of the surgery-first approach for skeletal Class III patients by establishing guidelines for a surgical occlusion setup in three dimensions.

The goal of surgery-first is to achieve normal skeletal relationship in three dimensions, skeletal symmetry in six planes of space and facial harmony. The surgical occlusion setup serves to foresee the tooth movements necessary to achieve an ideal occlusion after postsurgical orthodontic treatment. This is similar to the process that the orthodontist performs to correct any malocclusion of skeletal Class I, because skeletal deformity is corrected from the start. Because dental alignment, arch leveling and coordination, and incisor decompensation are deferred after surgery with the surgery-first approach, a major consideration for the surgical occlusion setup is to compensate for the space required for the dental movement. However, the previous guidelines have been rudimentary; that is, dental occlusion was proposed in the antero-posterior dimension only. For example, previous reports suggest the use of first molars as a guide for antero-posterior dental position^8,11,13,14^, which is similar to our guidelines. Because the compensation of horizontal mandibular relapse is planned for with a 2-mm overcorrection^8^, the Class II molar relationship is often set in our occlusion setup. Similarly, it is not surprising to note relatively large overjet (mean 4.4 ± 2.0 mm) in our occlusion setup as incisor decompensation is deferred after surgery.

The vertical dimension in our guidelines suggest deep overbite or posterior open bite for preventing bite opening from dental alignment and arch leveling after surgery as described previously^15,16^. The posterior open bite is easier to correct than anterior open bite after surgery; therefore, surgical occlusion setup with anterior open bite should be avoided. The posterior open bite is also helpful for correction of posterior cross bite from buccoversion of maxillary second molars, which is quite common in Class III malocclusion, due to unlocked occlusion.

Although a number of studies suggest no posterior cross bite at setup^7,11,13^, our guidelines in the transverse dimension emphasize the coordination of jaw midlines instead of dental arch to avoid positional jaw asymmetry. Posterior cross bite at setup appears in some cases of Class III malocclusion. It is mandatory to note whether it is skeletal or dental in nature. Orthodontic correction by archwire bending, inter- or intra-arch elastics, transpalatal arch or lingual arch is suggested in cases of dental cross bite or mild skeletal cross bite. On the other hand, segmental surgery or surgically assisted rapid palatal expansion is only indicated in patients with severe skeletal cross bite. Similarly, the posterior open bite at setup is also helpful for correction of posterior cross bite due to unlocked occlusion

It has been argued that stable occlusion at the time of surgery is important in postoperative stability^5–7,12^. However, the definition of stable occlusion varied between at least 3-point contact [7, 12] and stable posterior occlusion^5,6^. Our results indicate that stable occlusion can be achieved by occlusal contact on 5 to 6 teeth or occlusal contact on not only three segments but also on two or one (i.e., anterior) segments. Similarly, the posterior open bite at setup is also helpful to avoid severe postoperative occlusal instability from imperfect occlusal interdigitation, as described earlier.

In conclusion, the surgical occlusion for correction of skeletal Class III deformity using the surgery-first approach was set with positive overjet and overbite, Class II or I molar relation, and posterior cross bite and open bite. On average, there was occlusal contact on five to six teeth; most surgical occlusion setups had contact on three segments. The accuracy of the occlusion setup according the guidelines was excellent. Further studies are required to confirm the generalization of the guidelines to different types of dentofacial deformity.

## Patients and Methods

The study was conducted in accordance with the World Medical Association Declaration of Helsinki on medical research ethics. The approval of the study was granted by the Ethics Committee for Human Research at the Chang Gung Memorial Hospital in Taoyuan, Taiwan. Patients were included in the study if they met the following criteria: age ≥ 18 years; diagnosed with skeletal Class III deformity (ANB ≤ 0°) and no significant facial asymmetry (Menton deviation < 4 mm); surgical occlusion setup and computer-assisted surgical design conducted by a single experienced orthodontist; and received Le Fort I 1-piece osteotomy and bilateral sagittal split rotational setback with or without genioplasty using rigid internal fixation, and no intermaxillary fixation after surgery at Chang Gung Craniofacial Center, Taoyuan, Taiwan. Patients with one or more of the following criteria were excluded: a genetic syndrome or other congenital deformity; history of facial bone surgery; or mental retardation. A total of 53 patients were eligible for the study and were invited to participate.

### Guidelines for initial surgical occlusion setup

In the setup of surgical occlusion, it is important to decide (1) where to place the teeth antero-posteriorly, vertically, and transversely; and (2) how to position these teeth or tooth segments either surgically or orthodontically in order to achieve facial and dental esthetics, and maximize the efficacy and speed of treatment (i.e., simplify the orthodontic treatment after surgery). The guidelines used for occlusion setup are detailed below.

#### Sagittal relationship

When conducting a surgery-first approach, incisors cannot be used as a guide for antero-posterior jaw positioning (i.e., incomplete horizontal skeletal correction), unlike classic surgical-orthodontic treatment, in which incisor decompensation is performed before surgery. Instead, molars are the guide to antero-posterior jaw positioning. The molar relationship can be either Class I, II or III, depending on the tooth number ahead of molars. When there is a need for compensation of horizontal skeletal relapse, 2 mm overcorrection is often designed.

#### Vertical relationship

Because dental alignment and arch leveling is deferred after surgery with the surgery-first approach, a major consideration for the occlusion setup in the vertical dimension is to compensate for the space required for the dental alignment and arch leveling. Since dental alignment and arch leveling lead to proclination of the lower incisors and downward rotation of the mandible, thus decreasing the incisor overbite, the occlusion is set in deep overbite or posterior open bite. However, insufficient chin throat length (i.e., excessive horizontal skeletal correction) appears in some cases following bimaxillary rotational setback surgery^8,17^. It is mandatory to increase the posterior open bite by closing rotation of the osteotomized mandibular segment (i.e., distal segment), with the axis of rotation being the mandibular canines or premolars. However, the amount of posterior open bite resulting from this closing rotation should be within the limit of orthodontic tooth movement (<10 mm)^8^.

#### Transverse relationship

**Figure 1:**
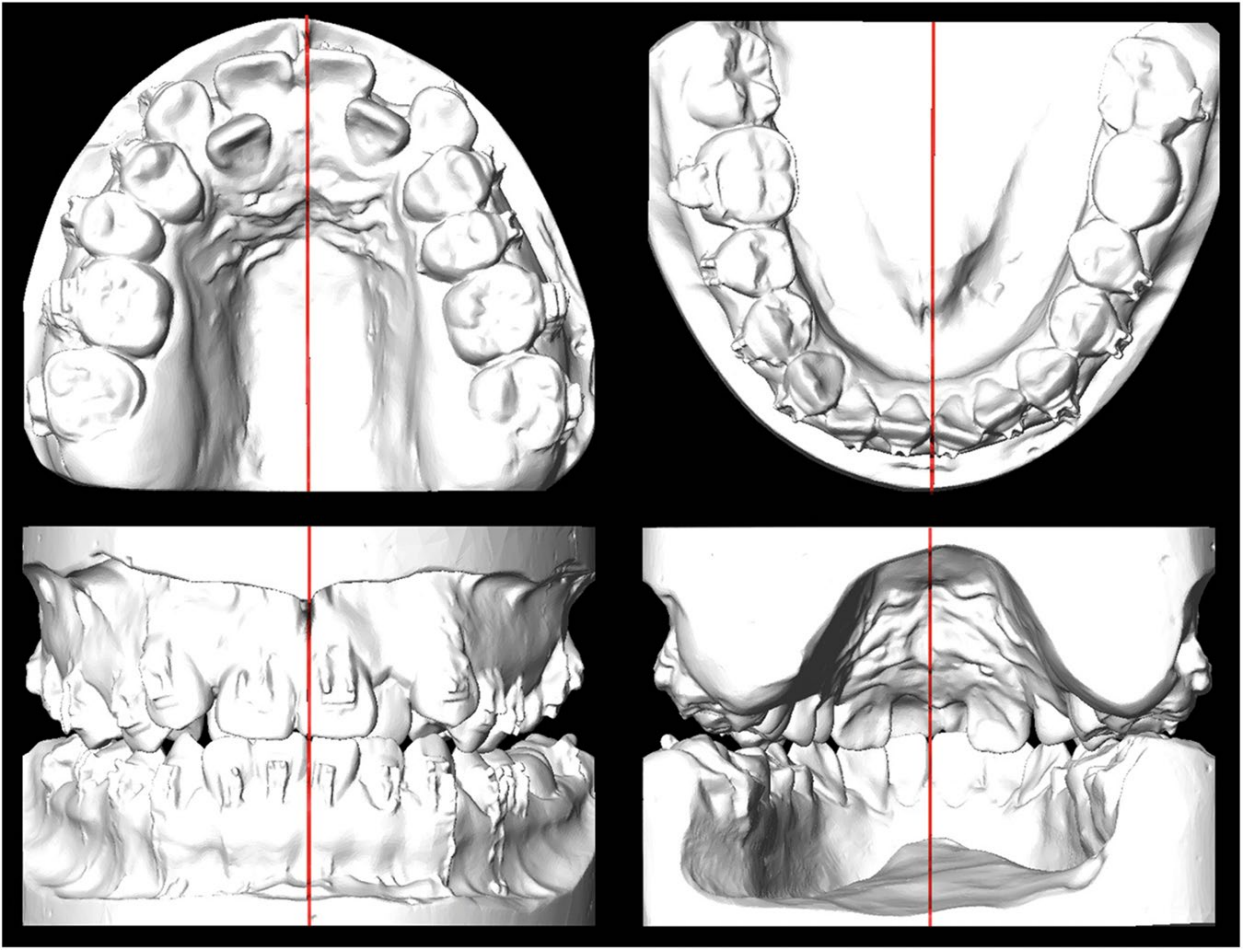
Dental casts showing surgical occlusion of a patient. (Top) Maxillary (left) and mandibular (right) casts; red indicates midlines. (Bottom) Surgical occlusion showing the coordination of the maxillary and mandibular jaw midlines; red indicates midlines.

Because arch coordination is deferred after surgery with the surgery-first approach, the occlusion setup in the transverse dimension often poses a significant challenge. A major consideration for the surgical occlusion setup in the transverse dimension is to achieve jaw symmetry. To prevent positional asymmetry, the maxillary and mandibular jaw (alveolar base) midlines must be coincident or close to it (Fig. 1).

#### Stable occlusion

Because dental alignment, arch leveling and coordination are deferred after surgery with the surgery-first approach, perfect occlusal interdigitation often cannot be achieved due to occlusal interference when performing model setup. To avoid severe postoperative occlusal instability, orthodontists can remove occlusal interference by simple occlusal adjustment in mild cases and opening the bite in more severe cases. Orthodontists can also perform limited orthodontic treatment (i.e., dental alignment) before surgery to remove severe occlusal interference precluding stable surgical occlusion.

#### Dental midline

Dental alignment is deferred after surgery with the surgery-first approach, therefore the surgical occlusion is sometimes set with dental midline off due to dental arch asymmetry. That is, surgical occlusion is set as coordination of jaw midlines instead of dental midlines.

### Contact distribution of surgical occlusion

One month before surgery, the patient’s maxillary and mandibular dental casts were scanned with a 3-dimensional (3D) laser surface scanner (3Shape, Copenhagen, Denmark). Surgical occlusion was then set according to the guidelines described above (initial occlusion setup) and scanned with the same 3D laser surface scanner.

**Figure 2:**
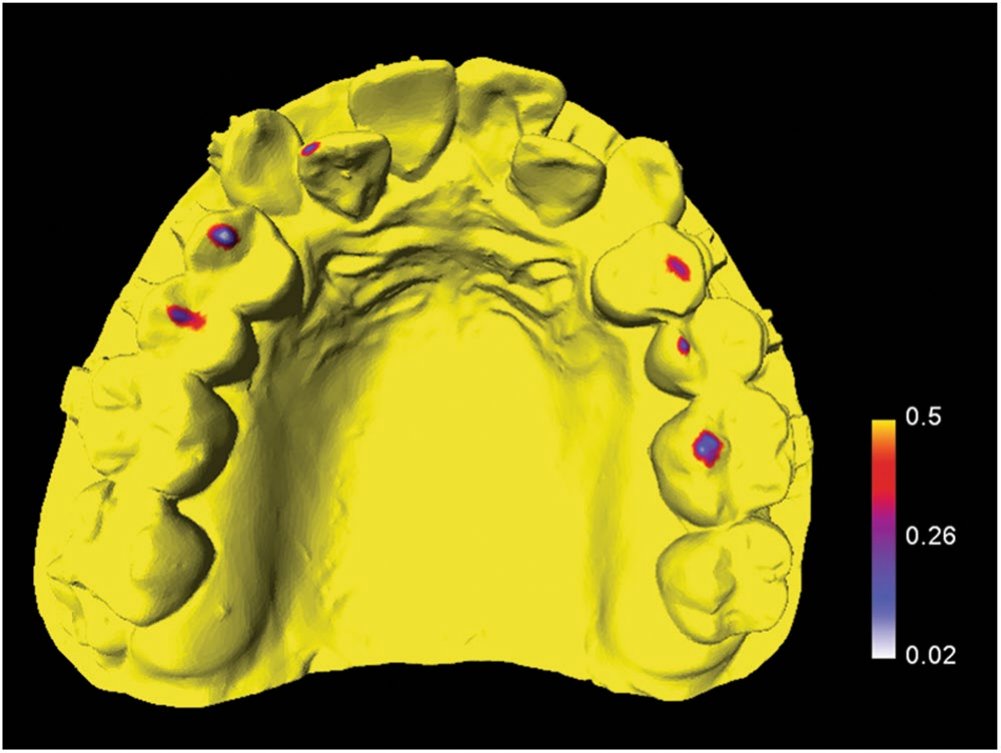
Maxillary dental cast of a patient showing surgical occlusal contact on three segments and six teeth.

Contact distribution of the surgical occlusion was measured using AVIZO version 7.0 software (FEI, Mérignac, France). The occlusal contact was defined as interocclusal distance being 0.5 mm or less and projected to the maxillary arch. The maxillary arch was divided into three segments: anterior, posterior right, and posterior left. Contact distribution was categorized as contact on three segments (anterior, posterior right, and posterior left), two segments (anterior and posterior right, anterior and posterior left, or posterior right and posterior left), or one segment (anterior, posterior right, or posterior left). Position of tooth contact (anterior, premolar, or molar) was also recorded (Fig. 2).

### Characteristics and accuracy of surgical occlusion

To determine the characteristics and accuracy of the surgical occlusion setup, one month before surgery cone-beam computed tomography of the head and neck was performed for each patient using an i-CAT 3D Dental Imaging System (Imaging Sciences International, Hatfield, Pa.). Patients were scanned during wakefulness with the following parameters: 120 kVp, 0.4 × 0.4 × 0.4-mm voxel size, 40-second scan time, and 16 × 16-cm field of view. The patient’s head was positioned with the Frankfort horizontal plane parallel to the ground. Throughout the scan, patients were instructed not to swallow, to keep their mouth closed, and to maintain a centric occlusion bite. Images were stored in Digital Imaging and Communications in Medicine format and rendered into volumetric images using Simplant OMS (Materialise, Leuven, Belgium). For a more accurate dental surface, the dental arches were replaced by scanned dental cast images. After defining Le Fort I and bilateral sagittal split osteotomy planes, the head images were reoriented according to the clinical measurements and cranial symmetry as well as Frankfort horizontal plane.

**Figure 3:**
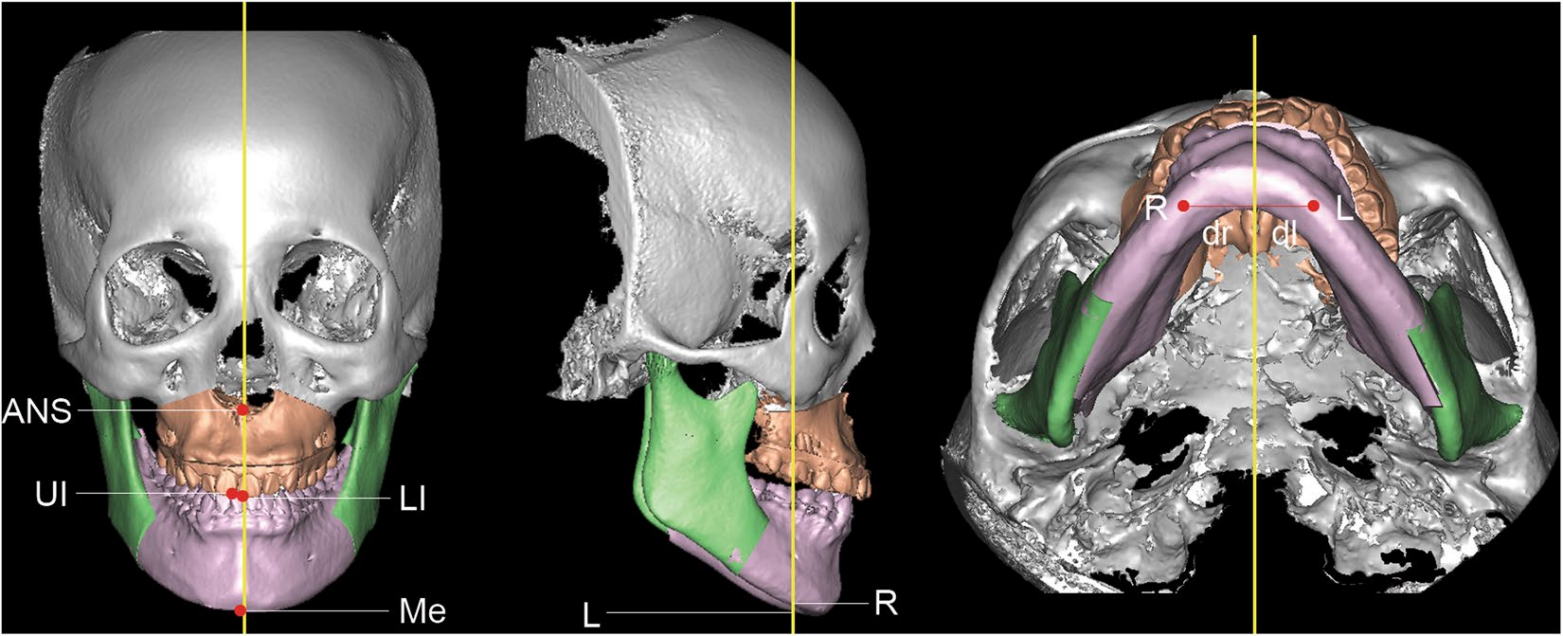
Computed tomography images used for linear and angular measurements of jaw symmetry. (Left) Horizontal deviation for maxilla (anterior nasal spine and upper incisor) and mandible (lower incisor and menton); ANS, anterior nasal spine; UI: upper incisor; LI: lower incisor; Me: menton. (Center) Vertical discrepancy for mandible (distance between point R and point L); point R: inflection point of mandibular body at the level of right mental foramen; point L: contralateral corresponding point of mandibular body on the left side. (Right) Horizontal discrepancy for mandible (distance discrepancy between dr and dl); dr: distance from point R to the facial midsagittal plane; dl: distance from point L to the facial midsagittal plane.

**Figure 4:**
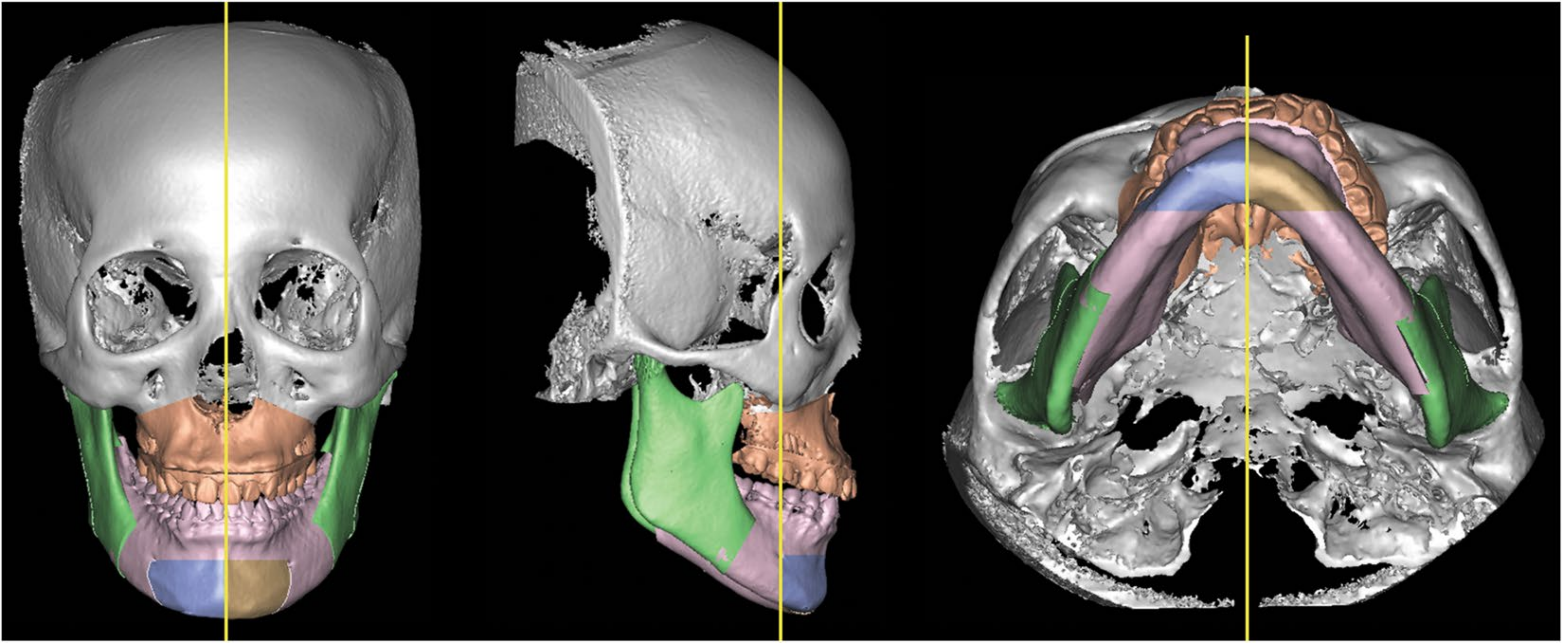
Computed tomography images used for volumetric measurements of jaw symmetry (volumetric discrepancy between right and left mandibular body).

Before virtual simulation, the surgical occlusion was transferred by moving the mandible to the fixed maxilla. Subsequently the maxillary and mandibular osteotomized segments were then moved as one united maxillo-mandibular complex according to the planning principles while maintaining the surgical occlusion^8^. That is, 3D simulation moved the maxillo-mandibular complex in the three dimensions until a normal jaw relationship and symmetry was achieved. When there was any significant skeletal deformity (often excessive horizontal skeletal correction or positional asymmetry due to incorrect initial occlusion setup), a new surgical occlusion was then set until deformity was corrected (final occlusion setup). The accuracy of the surgical occlusion was thus determined by calculating the incidence of the two setups. Virtual outcome regarding jaw relationship (i.e., ANB angle) and symmetry (i.e., midline deviation and mandibular body symmetry) were also calculated (Figs 3 and 4).

Characteristics of the surgical occlusion were assessed using linear measures to depict overjet and overbite, buccal overjet and overbite on second molars, and dental midline discrepancy. Angle molar relation (Class I, II, or III) was also recorded.

### Error of study

The error of the method was assessed through repetitive measuring of 10 randomly selected patients at least 1 week apart by the same investigator. The measurement error, evaluated using intraclass correlation coefficient (ICC), was excellent (mean ICC, 0.997; 95 percent confidence interval, 0.969 to 0.999).

### Statistical analysis

Data were analyzed using the statistical package SPSS (Version 17.0; SPSS, Inc., Chicago, Ill.). Unless otherwise specified, data are presented as the mean ± standard deviation (SD). Statistical analysis was carried out using the paired t-test, the Wilcoxon signed ranks test, the Mann-Whitney U test, or the chi-square test. All tests were two-tailed, with statistical significance set at p < 0.05.

### Data availability

The datasets generated during and/or analyzed during the current study are available from the corresponding author on request.
